# Psychometric properties of public trust in Covid-19 control and prevention policies questionnaire

**DOI:** 10.1186/s12889-022-14272-9

**Published:** 2022-10-24

**Authors:** Riaz Alaei Kalajahi, Mohammad Saadati, Saber Azami Aghdash, Ramin Rezapour, Mehdi Nouri, Naser Derakhshani, Koustuv Dalal

**Affiliations:** 1 Department of Health Management and Economics, School of Public Health, Tehran University of Medical Sciences, Tehran, Iran; 2 Department of Public Health, Khoy University of Medical Sciences, Khoy, Iran; 3 Tabriz Health Services Management Research Center, Health Management and Safety Promotion Research Institute, Tabriz University of Medical Sciences, Tabriz, Iran; 4 Student Research Committee, Tabriz University of Medical Sciences, Tabriz, Iran; 5 Health Management and Economics Research Center, Health Management Research Institute, Iran University of Medical Sciences, Tehran, Iran; 6 School of Health Sciences, Div. of Public Health Science, Mid Sweden University, Sundsvall, Sweden

**Keywords:** Trust, COVID-19, Policy, Management, Prevention

## Abstract

**Background:**

Public trust is a crucial concept in the COVID-19 pandemic, which determines public adherence with preventive rules as a success factor for disease management. This study aimed to develop and validate a tool to measure public trust in COVID-19 control and prevention policies (COV-Trust tool).

**Methods:**

This is a psychometric study that was conducted in 2020 (March-August). A primary tool was developed through literature review, in-depth interviews with experts and expert panel meetings. Content and construct validity was evaluated using content validity index (CVI) and content validity ratio (CVR) indexes and exploratory and confirmatory factor analysis, respectively. Cronbach α coefficient was calculated to determine the internal consistency.

**Results:**

A 28-item questionnaire with seven factors was developed. Factors included macro policy-making and management of pandemic, pandemic control policies implementing at all levels and their effectiveness, providing protective equipment and medicine for hospitals and public, prevention of negative socio-economic consequences of the pandemic, public participation, informing and public education and public behavior. The questionnaire reliability was calculated to be α = 0.959. Based on the experts’ opinion, tool content validity was estimated to be CVR = 0.73, CVI = 0.89. RMSEA = 0.07 revealed a good model fit as the confirmatory factor analysis results for the tool.

**Conclusion:**

COV-Trust tool is a well-fit tool to be used during this pandemic for improving policies effectiveness and could be used in similar situations as it determines the success of public health interventions.

**Supplementary Information:**

The online version contains supplementary material available at 10.1186/s12889-022-14272-9.

## Highlights

Investigating public trust during the outbreak of Covid-19 is of utmost important for both research and policy making perspectives.

COV-Trust tool (Seven factors and 31 items) was developed and validated.

COV-Trust tool comprehensively assesses public trust in various aspects of policies and their determinants and can provide valuable information for promoting the effectiveness of policies.

**Supplementary Information:**

The online version contains supplementary material available at 10.1186/s12889-022-14272-9.

## Introduction

The World Health Organization (WHO) identified COVID-19 as an epidemic in 2020 and considered it worrying and high-risk for countries with vulnerable health systems [1]. This is a serious threat to public trust in the government and the health system, leading to considerable negative consequences if appropriate action is not taken. Trust is one of the Achilles heels of governments and health systems [2]. Trust is the basis of the relation between people, the health system, and health providers. Patients often disclose their personal information and are sure that it will remain confidential[3]. To achieve this level of trust, health care systems should convince people that patients’ interests are more important than their personal or financial interests [4]. However, studies have shown that trust in health systems has declined over the past fifty years or more [5].

Public trust is an important concept that refers to the degree of public confidence and belief in the ability and competence of the health system to meet the needs and response ideally [6]. The higher level of trust, the easier to manage the unfavourable effects of an epidemic which are largely controlled by some health intervetions such as hand washing, staying home, and travel restrictions [6–8]. Public reactions to governments’ restrictive policies may depend on the level of their trust in health policymakers. However, little information is known about the effect of trust on adherence to health and safety regulations [9]. Most countries have taken several measures against this pandemic, from severe repression methods such as quarantine, forced social distancing, schools and unnecessary economic businesses and activities closures [10]. Public trust is more crucial when health rules require the contribution and cooperation of all members of the community [11, 12]. The lesson learnt from the recent outbreak of Ebola in 2018–2019 suggests that public trust in each country’s health system is vital to safety measures establishment [7]. Trust not only determines the success or failure of a risk management strategy, but the selected policy can also affect public trust and determine the success rate of future policies [13]. Investigations in diseases similar to COVID-19 have shown that it is essential to recognize community responses and insights into controlling and prevention policies [14, 15]. Therefore, public trust during the outbreak of Covid-19 is as important as clinical, epidemiological, and genomic studies. Most studies on trust during COVID-19 have examined political, organizational, information or individual trust related to Covid-19 disease [11, 16–19]. Farzanegan et al. (2022) concluded that the high mortality rate due to COVID-19 was negatively associated with public trust in government [18]. COVID-19 is slightly different from infectious diseases as it is a prolonged disease with various types of variants that may need different policies to be well controlled. This situation is required a high level of public trust in the government and policies to reduce the negative consequences of the disease. Considering that public trust in Covid-19 control and prevention policies is one of the topics that have been less addressed and comprehensive tools have not been provided to measure it, this study aimed to develop and validate a public trust in Covid-19 control and prevention policies questionnaire.

## Methods

This is a quantitative study conducted to develop and validate public trust in Covid-19 control and prevention policies (COV-Trust tool) questionnaire (annex 1). The study was developed and conducted in Iran from March to August 2020.

### Item generation and selection:

To identify the questionnaire items, a comprehensive literature search was conducted through Scopus, PubMed and Web of Sciences databases using the keywords of COVID-19, Corona, trust, policy, public, and government. Retrieved articles were reviewed, and two research team members extracted preliminary items. Parallelly, in-depth interviews were conducted with five experts (n = 5) in the field to achieve more items. Experts were academics in field of Health Services Management (n = 2), Health Policy (n = 1), Psychology (n = 1), and Epidemiology (n = 1). Experts were selected through purposive sampling. As a result of these two steps, 41 initial items were generated. Members of the research team reviewed the initial list during a meeting, and after merging similar items and excluding the duplicates, 35 items remained.

Furthermore, an 1.5-hour expert panel session was held in the faculty in April 2020 and generated items were reviewed, and required editions were made in items content. Moreover, items were categorized into seven categories based on their content similarity. Experts included individuals with the related field of study such as Health Education (n = 1), Health Policy (n = 3), Health Services Management (n = 2), Epidemiology (n = 2), experts who were engaged in COVID-19 policymaking at the provincial level (n = 2).

### Content validity

To determine the content validity of the questionnaire, two methods were used, including qualitative (experts’ opinions and suggestions) and quantitative (content validity index (CVI) and content validity ratio (CVR) methods. At this stage, the content validity form was provided to the experts and they were asked to evaluate each item based on the four criteria of “simplicity”, “relevance”, “transparency” and “necessity” (Table 1).

**Table 1:** Content validity evaluation form

Score	CVI			CVR
	Transparency	relevance	Simplicity	Necessity
4	Completely transparent	Completely relevant	Quite simple	Necessary
3	Transparent, but requires minor changes	Relevant, but requires minor changes	Simple, but requires minor changes	Useful but not necessary
2	Requires some changes	Requires some changes	Requires some changes	not necessary
1	Not transparent	irrelevant	Not simple	

According to the experts’ opinions, the content validity index (CVI) and content validity ratio (CVR) were calculated for each questionnaire item and the whole questionnaire. CVR and CVI were used to assess the content validity quantitatively. To calculate the CVI, three criteria of simplicity, relevance and transparency were used for each item, and the CVI score was calculated by summing the agreeing scores for each item ranked third and fourth on the total number of experts. Items with a score above 0.79 were approved. To calculate the CVR, the criterion of necessity in a 3-part Likert scale was used and calculated based on the following formula.

$$\text{C}\text{V}\text{R}=\frac{\text{n}\text{E}-\text{N}/2}{\text{N}/2}$$

nE: Number of experts who have responded to the “necessary” option.

N = Total number of experts.

At this stage, experts’ inclusion criteria were related academic education, research experience on COVID-19 policy and management issues/ public trust, and managerial experience in the health system at the city level or higher since the onset of the COVID-19 pandemic.

### Construct validity

To evaluate the construct validity of the Covid-Trust measurement tool, the data were randomly divided into two groups of 395 (15 cases out of 805 were removed because of missing data). In the first set of data, exploratory factor analysis (EFA) and in the second set, confirmatory factor analysis was applied. Factors were extracted through principal component with varimax rotation. Eigenvalue > 1 and theoritical interpretability was used for factors extraction. Model fit was examined using X^2^, χ^2^ / df, root mean square error of approximation (RMSEA), Goodness of Fit (GFI), Tucker–Lewis index (TLI), Normed Fit Index (NFI), and comparative fit index (CFI) parameters. In examining the indices, if the result of X^2^ analysis is significant, it indicates that the model does not fit. Values ​​close to zero in RMSEA index indicate more fit of the model and TLI, NFI and CFI indices values more than 0.95 indicate a good model fit. For the GFI index, a cut-off point of 0.0.95 was reported [20].

### Reliability

Cronbach α coefficient was calculated to evaluate and determine the internal consistency of the questionnaire. The internal consistency of the items and scales of the questionnaire was assessed independently by calculating the Cronbach α coefficient for the whole questionnaire and each of its components.

### Sampling

Based on the number of questionnaire items (29 items) and the norm provided to determine the sample size of factor analysis (10 to 15 times the number of items), 805 samples participated in the study. The study statistical population included members of virtual networks (Whatssapp and Telegram) throughout the country which were the most popular in Iran. A convenience random sampling was used to select the samples. As channels wih countrywide members were selected and the online questionaire link was published in channels in 4 different days in a week including weekend, first, third and fifth days of the week. Data collection was done from May to july 2020.

### Statistical analysis

Cronbach α index was calculated to assess the reliability by the internal consistency method. Values ​​greater than 0.7 were considered as good reliability. To investigate the tool construct validity, exploratory factor analysis (EFA) was run and then confirmatory factor analysis (CFA) was used. Analyses were performed using SPSS 26 and Amos 26 software.

## Results

### Population characteristics

A total of 805 people participated in the study. Most of the participants in the study were female (53.5%) and had a bachelor’s degree (32.7%) and a master’s degree (32.5%). The majority of the participants were in the age group of 21 to 39 years (60.3%). In terms of income level, most people (60.1%) had declared a moderate-income level (Table 2).

**Table 2:** Demographic characteristics of the participants

Variables	Groups	Frequency	Percent
Age groups (years)	> 20	39	4.9
	21–39	481	60.3
	40–59	259	32.5
	60>	19	2.4
Gender	Female	373	53.5
	Male	323	46.4
Education	PhD	113	6.7
	Master	176	32.5
	Bachelor	301	32.7
	Diploma and lower	211	12.7
Job	Non-public sector staff	54	6.7
	Public sector staff	102	12.7
	Others	263	32.9
	Health care staff	262	32.7
	Self-employed	118	14.7
Economic status*	Moderate	482	60.1
	Poor	152	18.9
	Good	124	15.4
	Very poor	30	3.7
	Very good	14	1.7

### Content validity

Seven factors and 31 items were selected based on expert opinions. After reviewing experts’ opinions, content validity indices were calculated for all items and two items were excluded due to CVR score < 0.42 and CVI < 0.79. Finally, 29 items were analysed for construct validity.

### Construct validity

**Fig. 1:**
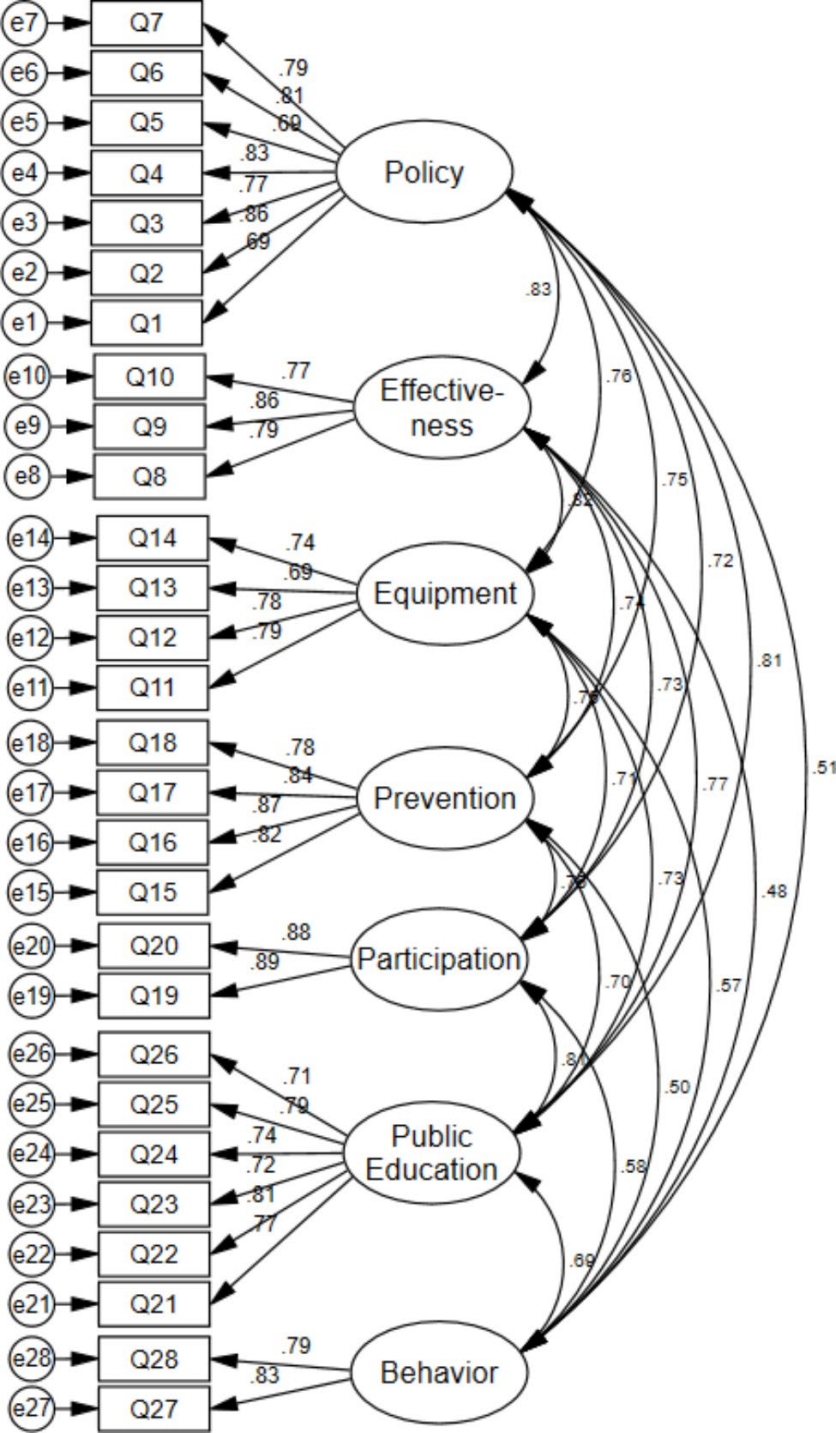
Measurement model of public trust in coronavirus prevention policies: factor loadings for 28 items and 7-factor model **Fit indices: Chi-square = 777.21, DF = 329, Chi-square to DF = 2.36, SRMR = 0.041, RMSEA = 0.059, GFI = 0.091, AGFI = 0.90, NFI = 0.91, IFI = 0.93, CFI = 0.94, TLI = 0.93**

Model fit indices in exploratory factor analysis confirmed the 28-item, seven-factor model (Table 3). One item was eliminated bacause of lower loading than 0.3 in EFA. Extracted factors include macro policy-making and management of pandemic, pandemic control policies implementing at all levels and their effectiveness, providing protective equipment and medicine for hospitals and public, prevention of negative socio-economic consequences of the pandemic, public participation, informing and public education and public behavior (Appendix 1). CFA approved the extracted factors with good model fit indices (Fig. 1). Also, the relationship between all items and components and the correlation between the seven components was statistically significant (P < 0.001) and the standardized regression weight of all items with related factors was more than 0.3.

**Table 3:** COV-Trust EFA and CFA results

Factors and Items		EFA*		CFA			
		**Loadings**	**Explained variance %**	**Loadings**	**Z-stat**	AVE**	**CR** ^**#**^
Policy	Q1	0.725	18.32	0.694	-	0.61	0.92
	Q2	0.762		0.857	15.89		
	Q3	0.739		0.774	14.45		
	Q4	0.660		0.828	15.40		
	Q5	0.665		0.694	13.04		
	Q6	0.636		0.808	15.05		
	Q7	0.622		0.786	14.66		
Effectiveness	Q8	0.548	13.87	0.794	-	0.65	0.85
	Q9	0.682		0.858	18.50		
	Q10	0.634		0.766	16.15		
Equipment	Q11	0.589	12.68	0.789	-	0.56	0.84
	Q12	0.757		0.777	15.96		
	Q13	0.775		0.690	13.92		
	Q14	0.528		0.736	15.01		
Prevention	Q15	0.729	9.66	0.819	-	0.68	0.90
	Q16	0.740		0.868	20.28		
	Q17	0.813		0.837	19.30		
	Q18	0.705		0.780	17.49		
Participation	Q19	0.593	6.69	0.894	-	0.79	0.88
	Q20	0.682		0.884	22.72		
Public Education	Q21	0.703	6.60	0.771	-	0.57	0.90
	Q22	0.699		0.812	17.14		
	Q23	0.672		0.722	14.91		
	Q24	0.473		0.737	15.27		
	Q25	0.654		0.786	16.48		
	Q26	0.517		0.706	14.51		
Behavior	Q27	0.808	5.99	0.831	-	0.65	0.79
	Q28	0.820		0.786	12.97		

### Reliability

Examining the internal consistency of questionnaire structures showed that all scales and subscales of the questionnaire have the minimum reliability standard (0.7) and Cronbach α coefficients were calculated to confirm the reliability of this questionnaire at the desired level (Table 4).

**Table 4:** Results of the internal consistency assessment (Cronbach’s alpha) of the questionnaire

Trust dimensions	MEAN ± SD	Cronbach's α
Macro policy-making and management of epidemic	38.03 ± 26.32	0.92
Pandemic control policies implementing at all levels and their effectiveness	35.06 ± 25.88	0.85
Providing protective equipment and medicine for hospitals and public	33.07 ± 24.88	0.84
Prevention of negative socio-economic consequences of the pandemic	25.70 ± 23.87	0.85
Public participation	31.86 ± 26.82	0.83
Informing and public education	40.79 ± 24.81	0.89
Public behavior	36.48 ± 25.35	0.78
**Total**	**34.43 ± 28.80**	**0.96**

## Discussion

The present study developed and validat ed public trust (COV-Trust tool) in the context of Covid-19 control and prevention policies. The COV-Trust tool was designed to measure trust in heath policies through 28 items in 7 components with the reliability of α = 0.959 and validity of CVR = 0.73, CVI = 0.89. Its components include macro policy-making and management of pandemic, pandemic control policies implementing at all levels and their effectiveness, providing protective equipment and medicine for hospitals and public, prevention of negative socio-economic consequences of the pandemic, public participation, informing and public education and public behavior. The exploratory and confirmatory factor analysis results showed good model fit and construct validity of this tool.

Trust is the cornerstone of the relationship between people and public institutions such as the health system. It has been introduced as a determining factor in public adherence to social and even personal health protocols [4, 12, 21]. Studies have shown that public trust in the health system has declined over the past decades [22] and the outbreak of the Coronavirus pandemic caused a significant threat in this regard [4]. During the pandemic, policies to control and prevent coronavirus disease, including social distancing, quarantine, using a mask, and handwashing, was recommended by the WHO, which required public adherence in their personal and social life [1]. Public trust in these policies is the weak point of this issue, and governments must make the necessary predictions about this issue since people trust in the policies adopted by governments and their adherence will determine the future of the Covid-19 pandemic [6, 13].

Prior studies have investigated the impact of political trust on the social behaviour of the public during the Covid-19 pandemic and have shown that the political trust was associated with public adherence to the required social behaviours during the pandemic, especially in non-pharmaceutical interventions [9, 16, 17]. Low public trust leads to people’s violations of quarantine rules and regulations [18, 23]. It is also true about the coronavirus vaccination, as evidence suggests that lack of trust in vaccines’ safety and efficacy has caused hesitancy among the public and health professionals[24]. Continuous assessment of public trust in this area will provide helpful information for policymakers to make necessary changes and reforms in current policies based on the people’s preferences.

The questionnaire, in the dimension of macro policy-making and management of pandemic (α = 0.906), assesses public trust in the integrity, credibility and competence of participants in the process [25] by asking the people on the use of scientific and up-to-date evidence, experts engagement, prioritization of vulnerable individuals and groups, as well as benchmarking and using the experiences of other countries. The second component of the questionnaire examines public trust in the implementation and effectiveness of policies at all national, regional and local levels (α = 0.778). This dimension measures the integrity of policies at all management levels of the country. Lack of coordination in policies and executive programs at national and local levels leads to low effectiveness [26]. Trust in the equitable supply and distribution of medical and protective equipment is one of the most critical issues in coronavirus control policies, which is assessed by the third dimension of this tool (α = 0.842). Coordination in the system of preparation and distribution of this equipment and its feedback at the community level will lead to a change in the level of public trust [27].

Prevention of pandemic negative socioeconomic consequences and Public behavior are the other two dimensions of the questionnaire, which includes topics that affect people’s lives especially vulnerable groups. It affects people peace of mind and their trust in the proper functioning of the government in this regard. In addition to creating social peace and preventing harmful movements of groups, it can increase people adherence to announced health guidelines [28, 29]. Public participation is another dimension assessed by the designed tool (α = 0.777). Public assurance of the government’s intention to use the local potentials and public participation in its policies to control the pandemic could facilitate the government [30]. Participation can occur in a range of activities and at different levels, from informing participation in planning and evaluation of policies [31]. Continuous monitoring of public trust in information channels and preventing inaccurate information dissemination is an issue assessed by the developed tool. The importance of informing and public education in the coronavirus pandemic and the involvement of different individuals and groups in this issue led to the creation of “infodemic” terminology [32]. Paving the way for the dissemination of accurate and precise information and gaining people trust in the determined paths is one of the necessities to increase trust, which results in increased people adherence to control and prevention policies, especially in the social aspect [19, 33].

The study has some limitations which should be considered when generalizing the results. This study was done during the pandemic, and the estimations may be affected by the peoples’ mental and psychological status. The study samples were individuals who could use online virtual networks, which may not represent the population well. Moreover, we did not use any external criterion instrument to assess the concurrent validity. Also, for reliability analysis, we did not use test-retest analysis.

## Conclusion

The COV-Trust tool developed in this study could be used as a standard tool by researchers and governments to independently assess public trust in Covid-19 control and prevention policies. This tool comprehensively assesses public trust in various aspects of policies and their determinants and can provide valuable information for promoting the effectiveness of policies.

## Electronic supplementary material

Below is the link to the electronic supplementary material.

Supplementary Material 1

## Data Availability

Interested author can contct Mohammad Saadati (saadati_m@khoyums.ac.ir, hcm.2020@gmail.com) for relevant data and information.

## Abbreviations

CFI: Comparative fit in.
CVI: Content validity index.
CVR: Content validity ratio.
GFT: Goodness of Fi.
NFI: Normed Fit Index.
RMSEA: Root mean square error of approximation.
TLI: Tucker–Lewis index.
WHO: World Health Organization.
