# Supplementary material for: Psychometric properties of public trust in Covid-19 control and prevention policies questionnaire
Source: BMC Public Health. 2022 Oct 24;22:1959. doi: 10.1186/s12889-022-14272-9 (PMC9589749; doi:10.1186/s12889-022-14272-9)
Supplement: Supplementary file 1 — Supplementary Material 1 [file 12889_2022_14272_MOESM1_ESM.docx]

**Annexure-1**

Public trust on Covid-19 control and prevention policies tool

| NO. | Item | Completely agree | Agree | No opinion | Disagree | Completely disagree |
| --- | --- | --- | --- | --- | --- | --- |
|  | I am confident that the top priority is given to public health while adopting preventive measures |  |  |  |  |  |
|  | I am confident that there are appropriate preventive measures and policies being implemented against Covid-19 pandemic nationally. |  |  |  |  |  |
|  | I am confident that scientific evidence and best practices has been/ are taken into consideration while providing preventive measures |  |  |  |  |  |
|  | I am confident in the capabilities and the accountability of the responsible directors/ managers/ decision-makers to face the pandemic |  |  |  |  |  |
|  | I am confident that NGOs and other civil societies are engaged in controlling the pandemic |  |  |  |  |  |
|  | I am confident that there are appropriate measures taken against disease to protect vulnerable individuals (e.g., aged groups, people with chronic diseases) |  |  |  |  |  |
|  | I am confident that there are enough resources dedicated and adequate authority delegated to the “National Committee for Fighting Covid-19 (NCFC)” to fight the disease |  |  |  |  |  |
|  | I am sure that all responsible bodies take the declared guidelines by NCFC into action properly |  |  |  |  |  |
|  | I am generally confident that the preventive measures result in the pandemic to cease or slow down |  |  |  |  |  |
|  | I am confident that there are adequate number of infrastructures and facilities available in my living area and in case of any deficiencies it will be dealt with immediately |  |  |  |  |  |
|  | I am sure that there is enough medical equipment available to be used in hospitals and health centers for patient care |  |  |  |  |  |
|  | I am sure that there are enough personal protective equipment (PPE) e.g., masks, face shields, gloves, gowns, etc. in hospitals |  |  |  |  |  |
|  | I believe not only there are enough PPE available for public e.g., masks, antiseptic, but also, they are proportionately distributed |  |  |  |  |  |
|  | I am sure that necessary drugs and medical equipment to take care of those suspected to infection at home |  |  |  |  |  |
|  | I am confident about the supply of basic food products at market if the pandemic continues to exist for longer times |  |  |  |  |  |
|  | In case of lasting quarantine and social distancing, I am sure that vulnerable families (economically) will be strongly supported |  |  |  |  |  |
|  | I am sure that favorable policies have been adopted to avoid harsh economic down-turns |  |  |  |  |  |
|  | I am confident that preventive measures to avoid social harms due to Covid-19 (i.e., increased suicide rates, in-house violence especially those affecting children and female, increased rate of divorces, phobia, stress, and crime) are prepared |  |  |  |  |  |
|  | To prevent and control mental health sufferings (especially within infected individuals, hospital staff and their families, families who are affected by covid due to an infected or a lost family member), I believe, necessary measures have been taken into consideration |  |  |  |  |  |
|  | I am confident that there is a positive climate established for a systematic and programmatic community engagement to control the pandemic |  |  |  |  |  |
|  | am confident that the state strongly supports the communities to implement measures and policies adopted to face the pandemic |  |  |  |  |  |
|  | I am confident that health educations for public are provided according to their needs |  |  |  |  |  |
|  | I am confident that that the educational contents provided for public are extracted from recent scientific updates on the disease |  |  |  |  |  |
|  | I am confident that families who care for patients at home or the patients themselves will receive the necessary educations for homecare |  |  |  |  |  |
|  | I am confident that information provided by NCFC are about death tolls and cases of new infections are real or at least close to reality |  |  |  |  |  |
|  | Enough attention, I am confident, is paid for conducting research on drugs and vaccines in academic premises and universities |  |  |  |  |  |
|  | am confident that the application of measures to prevent the spread of misinformation and rumors are ensured |  |  |  |  |  |
|  | It seems that people are aware of the necessities of proper mask-wearing |  |  |  |  |  |
